# Resampled Efficient Frontier Integration for MOEAs

**DOI:** 10.3390/e23040422

**Published:** 2021-03-31

**Authors:** David Quintana, David Moreno

**Affiliations:** 1 Department of Computer Science, Universidad Carlos III de Madrid, 28911 Leganés, Spain; 2 Department of Business Administration, Universidad Carlos III de Madrid, 28903 Getafe, Spain; jdmoreno@emp.uc3m.es

**Keywords:** porfolio optimization, MOEA, finance, robustness

## Abstract

Mean-variance portfolio optimization is subject to estimation errors for asset returns and covariances. The search for robust solutions has been traditionally tackled using resampling strategies that offer alternatives to reference sets of returns or risk aversion parameters, which are subsequently combined. The issue with the standard method of averaging the composition of the portfolios for the same risk aversion is that, under real-world conditions, the approach might result in unfeasible solutions. In case the efficient frontiers for the different scenarios are identified using multiobjective evolutionary algorithms, it is often the case that the approach to averaging the portfolio composition cannot be used, due to differences in the number of portfolios or their spacing along the Pareto front. In this study, we introduce three alternatives to solving this problem, making resampling with standard multiobjective evolutionary algorithms under real-world constraints possible. The robustness of these approaches is experimentally tested on 15 years of market data.

## 1. Introduction

Asset allocation has traditionally been one of the most important topics in finance. There have been a number of efforts to identify the best combinations of financial assets, but the most popular one, Modern Portfolio Theory, is based on the seminal work of Markowitz [1,2]. According to this framework, the problem can be modeled as a multiobjective optimization problem where the decision-maker tries to identify the best portfolios according to two competing dimensions: risk an return. The best asset combinations would define a Pareto front, known in this context as the efficient frontier. The portfolios that comprise this efficient frontier are equally good, in the sense that none of them dominates any of the rest. For each level of risk, there are no alternatives that offer a higher return or, for each return level, there are no portfolios that offer a lower risk. This means that the choice of one of them would be based on the preferences of the decision-maker, unless there is a risk-free asset available.

Under the ideal conditions defined by Markowitz, this optimization problem can be solved using quadratic programming. However, once we start considering additional real-world constraints, the problem becomes more mathematically complex, and tackling it using traditional methods requires increasingly sophisticated instruments, like conic optimization or sparse mean–variance portfolio modeling, and simplifying assumptions [3,4,5,6]. At one point, the problem becomes so complicated that both researchers and practitioners often start using metaheuristics from the field of evolutionary computation like genetic algorithms, differential evolution or evolution strategies, to name a few. Among these, multiobjective evolutionary algorithms (MOEAs) are progressively gaining traction [7,8,9,10] as they have the ability to return the whole efficient frontier in one go, compared to the more traditional mono-objective ones, which require gridding approaches to obtain it. Still, real-world constraint management with these algorithms is a challenging matter [11]. For a detailed discussion on the state-of-the-art of mean–variance portfolio optimization, the interested reader is referred to recent surveys by Kalayci et al. [12] and Ferreira et al. [13].

Despite the instrument used, this optimization problem requires at least two sets of parameter: future asset returns and variance–covariance matrix. Given that this information is not available, we rely on estimates, which are subject to estimation errors. This means that structure of the actual best-performing portfolios might be very different from the ones obtained from the output of the optimization process or, looking at the problem from a different angle, that the observed risk/return profile of the selected portfolios might be far from the anticipated one. In fact, several authors, such as Kritzman [14], showed that the portfolio misallocation could be very large, even higher than half of the portfolio composition. This creates a robustness problem already discussed by authors like Jorion [15].

The efforts to limit the impact this problem have led to the development of robust portfolio optimization methods. Among them, we can identify two major lines of work in the finance literature, one that optimizes for the worst-case scenario, and another that simultaneously considers a number of synthetic ones. Within the latter, which is where we place the contributions of this study, we would highlight the resampled efficiency technique, introduced by Michaud and Michaud (MM) [16,17], which identifies efficient frontiers for different scenarios and combines them to obtain a robust efficient frontier.

The highly influential solution suggested by MM, to obtain the final solution from the set of Pareto fronts, is effective, but has two limitations. The first one is the fact that, under some real-world constraints, it might result in unfeasible solutions. The second has to do with the impossibility of using it with the output of most MOEAs, as it requires them to have exactly the same number of portfolios along the efficient frontier and regular spacing among them. These traits, despite being desirable goals of multiobjective optimization, cannot always be guaranteed.

The field of evolutionary multiobjective optimization has developed a number of specific solutions for the problem of robust portfolio optimization. These usually require special-purpose algorithms or significant adaptations of the fitness functions or evolutionary operators.

Among them, we could mention contributions like [18], which introduces a time-stamped resampling strategy that extends the basic two-objective framework, adding a third explicit robustness objective, or [19], focused on the multiperiod version of the problem. In this latter case, the authors propose the integration of an anticipatory learning method using Kalman Filters (ASMS-EMOA).

Quintana et al. [20], also present another variation of SMS-EMOA, a robustness-based S-metric selection evolutionary multiobjective optimization algorithm (R-SMS-EMOA) for robust portfolio optimization. This algorithm adjusts the optimization process, focusing it on the most stable regions of the search space to mitigate portfolio implementation risk. This is done according to user preference information in terms of the desirable degree of robustness in the solutions. More recently, Solares et al. [21] suggest dealing with uncertainty regarding parameters by defining them as ranges. They then adapt the Multiobjective Evolutionary Algorithm Based on Decomposition (MOEA/D) to handle this information and test the system on a constrained optimization framework, where they consider budget, non-negativity and the bounds on individual stocks constraints.

In this paper, we introduce three novel combination approaches that are compatible with the output of the standard versions of MOEAs so that resampling is easily implemented with any off-the-shelf implementation, without the need to alter the basic algorithm or to rely on complicated fitness functions.

The rest of the paper is structured as follows. First, we introduce the suggested approaches. This is followed by a description of the experimental approach used to test them, including a detailed description of the optimization problem, the algorithm used to solve it, and an introduction of the sample. Then, we report the experimental results, and we devote the last section to providing a brief summary and reporting the main conclusions.

## 2. Suggested Approaches

According to Modern Portfolio Theory, the standard problem of financial portfolio optimization requires the meeting of two opposing objectives, the maximization of expected portfolio return $E(R_{p})$, and the minimization of its risk, which is usually defined in terms of the portfolio variance, $\sigma_{p}^{2}$, or standard deviation. In the basic framework, the optimization is subject to two constraints, full investment requirement (all the funds should be distributed among the assets) and limiting the possibility of shorting assets. That is

- Minimize risk:
  (1) $$\sigma_{p}^{2}=\Sigma_{i=1}^{n}\Sigma_{j=1}^{n}w_{i}w_{j}\sigma_{ij}$$
- Maximize return:
  (2) $$E\left(R_{p}\right)=\Sigma_{i=1}^{n}w_{i}\mu_{i}$$
- Subject to:
  (3) $$\Sigma_{i=1}^{n}w_{i}=1$$
  (4) $$0\leq w_{i}\leq 1;i=1...n$$
  where *n* represents the number of assets that might be included in the portfolio, $\mu_{i}$ is the expected return for asset *i*, $\sigma_{ij}$ is the covariance of returns between asset *i* and *j*, and $w_{i}$ is the weight of asset *i* in the portfolio.

As we mentioned in the introduction, this is often solved by turning it into a mono-objective problem, using a grid to specify a number of equally spaced target returns, and minimizing risk for each of them using using quadratic programming. Alternatively, the problem might be solved using a very similar approach, which identifies the efficient frontier, optimizing a utility function for different levels of risk tolerance.

This basic representation of the problem can be extended to include additional real-world constraints, like limits to the maximum and minimum weight of any individual asset, or the maximum or minimum number of assets that can be part of the solution simultaneously.

- Investment limits: weights $w_{i}$ must be defined in the interval [$lim_{inf}$, $lim_{sup}$], where
  (5) $$lim_{inf}\leq w_{i}\leq lim_{sup}$$
- Cardinality constraints: there is a minimum $C_{min}$ and maximum $C_{max}$ number of assets that can be part of a portfolio ($w_{i}\neq 0$).

Once we start adding these constraints, the problem becomes harder to solve using traditional methods, and metaheuristics start becoming the instrument of choice.

This optimization relies nf two key parameters, the estimates for the expected returns and the variance–covariance matrix. Depending on the sophistication of investors, these might be obtained in different ways, but the most common solution consists of using historical data over a particular time window. Given a number of time periods, the forecast for future returns is the average of the historical ones, and the future variance–covariace matrix will be the one observed over the period. As mentioned, there are other alternatives, like Black–Litterman [22], that require the input of financial analysts, or the Shrinkage covariance estimation by Ledoit and Wolf [23].

A complete focus on the expected return and variance–covariance matrix might lead to solutions that are very sensitive to estimation errors. For this reason, Michaud and Michaud [16,17] introduced a resampling method that considers several scenarios, making the solution more robust. In order to generate reasonable scenarios, they recommend using bootstrap to generate synthetic series of returns. Once we obtain these, we only have to compute the expected returns and the variance–covariance matrix, and optimize for them, achieving a new efficient frontier. If we repeat the process a number of times, we obtain several estimates for the future efficient frontier, which we combine to obtain the final solution.

**Figure 1:**
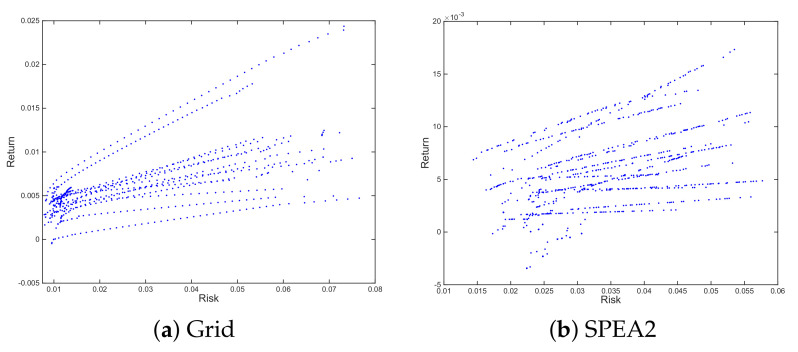
Illustration of general behavior of Efficient Frontier for different scenarios. Standardized mono-objective grid optimization, as required by Michaud & Michaud (Grid) vs. MOEA (SPEA2). Panels based on same time window, but with different resamples.

These authors suggest tackling the multiobjective optimization problem using a grid and turning it into several mono-objective ones. This entails obtaining the limits of the efficient frontier, the minimum-risk portfolio and the maximum-return one, and dividing the range of returns into a user-defined number of equidistant intervals. The efficient frontier for each scenario is then defined as the set of portfolios that minimize risk for the different reference return levels, as illustrated in Figure 1, panel a. Another alternative, also discussed by MM, consists of selecting a number of values along the range of potential risk tolerances, from total risk aversion to total risk indifference, and maximizing utility for each of them. This process results in an output similar to the one illustrated in Figure 1, panel a.

Given that the returns for the portfolios that comprise the efficient frontiers for the all scenarios are aligned, these authors suggest combining them, averaging the weights of the portfolios that are placed in the same position (or have the same rank), under the rationale that they tend to cater to investors focused on similar regions of the efficient frontier. That is, the weights of all minimum-risk portfolios for the synthetic scenarios are averaged by asset to obtain the final resampled minimum-risk portfolio. Then, the portfolios with the second lowest risk across the efficient frontiers for all the synthetic scenarios are selected, averaging their weights by asset, and the resulting portfolio is used as the second portfolio in the final resampled efficient frontier, etc. This results in a set of average portfolios that define the robust resampled efficient frontier. Please note that the same process is applicable regardless of whether the association of portfolios is based on return or risk aversion.

In case the efficient frontiers for the synthetic scenarios have either different numbers of portfolios, or very irregular spacing, the process becomes problematic for two reasons: averaging might not be possible, or it might result in a combination of portfolios that have little to do in terms of their risk/return profile, and does not make any financial sense.

This is the reason this methodology poses a problem in cass where the optimization for the synthetic scenarios was made using MOEAs. Given their nature, it is often the case that neither the number of portfolios, nor even regular separation of consecutive portfolios along the efficient frontier, can be guaranteed. The scale of the problem depends on the algorithm, as both aspects are the goals of multiobjective optimization and better algorithms will be less prone to major irregularities in these two fronts, but the structure is likely to be similar to the one depicted in Figure 1, panel b, which was obtained by optimizing for 20 resampled scenarios using SPEA2 [24].

This limitation makes the need for alternative techniques apparent. In this section, we discuss three approaches to overcome it.

### 2.1. Approach 1

The first possibility is an approximation of the basic approach, which starts by fitting a polynomial function to the complete set of resampling efficient frontiers. This proxies the mean efficient frontier with a continuous function. Regarding the polynomial order, 2 is likely to be a good starting point, but it could be higher depending on the structure of the data.

**Figure 2:**
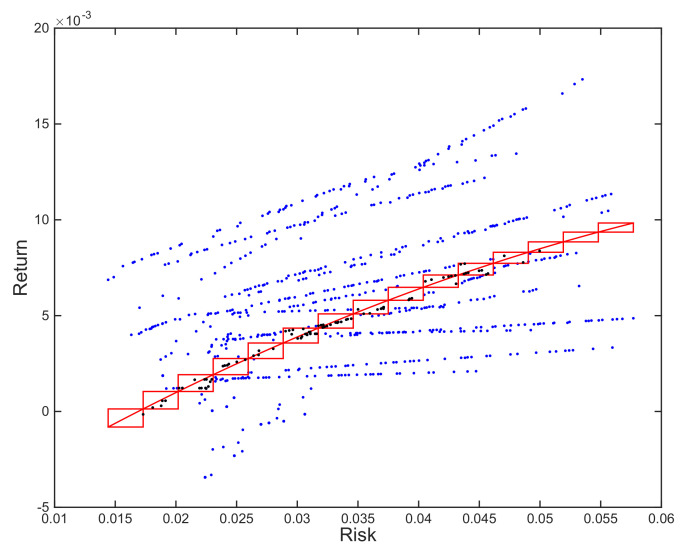
Illustration of approach 1 on 20 resampled frontiers optimized using SPEA2 and an order 2 polynomial trendline. Boxes defined by reference points along the polynomial trendline that serve as corners. Portfolios in final solution obtained averaging the composition of portfolios in the same box (in black).

Then, we identify the range of returns, minimum and maximum risk among all portfolios, and divide them into a user-defined number of linearly spaced values. The polynomial function is subsequently used to obtain the returns for these risks, using every pair of consecutive datapoints to define a box. The result of this process is a string of boxes where the upper-right corner of the one on the left is connected to the bottom-left corner of the one on the right. This is illustrated in Figure 2.

The next step is averaging the composition of the portfolios included in each box—black dots in the Figure—obtaining as many portfolios as boxes with contents as a result. The final step involves removing any dominated portfolios, leaving the resulting set as the expected efficient frontier.

This approach has the advantage of providing average portfolios near the estimate for a mean efficient frontier, but it also has limitations in terms of the number of elements in the solution. The number of intervals defines an upper bound, but nothing guarantees that there will be portfolios in every box. A second limitation has to do with the feasibility of the solutions. Averaging portfolios might be problematic once we start dealing with investment limits or cardinality constraints, as we commented for the MM method. The fact that the portfolios combined in each box meet the constraints at an individual level does not guarantee that the average one will and, therefore, a significant portion of the final solution might be unfeasible.

However, the second limitation could be mitigated using a variation that includes a reconstruction algorithm to convert unfeasible solutions into feasible ones. This kind of operator introduces biases and might result in dominated portfolios, hence the need to add an additional step to limit the final portfolio selection to the set of non-dominated ones.

### 2.2. Approach 2

The limitation discussed before can be tackled with a similar strategy that, instead of averaging feasible solutions, selects promising candidates among a large set of feasible ones. In this case, the starting point is the same. The dataset is resampled to generate synthetic scenarios, which are optimized so that we obtain as many efficient frontiers as scenarios, and a polynomial function is fitted to the resulting combined set of solutions.

Then, we just have to select a number of *v* linearly spaced values along the risk axis, obtain the expected returns using the polynomial functions and, for each of these datapoints, find the portfolio with the most similar risk/reward profile among the complete set.

There is, however, something to bear in mind. The mentioned set consists of porfolios that were optimized for a single scenario. The fact a portfolio happens to be close to the mean reference efficient frontier defined by the polynomial function does not make it robust. Its mean expected risk and return might be far from the observed one for the synthetic scenario that we obtained it from. Having said that, even though the individual portfolios might not be robust, the combined resampled efficient frontier as a whole is more likely to, as it consists of portfolios optimized for different scenarios that might react differently to deviations between the expected parameters and the real ones.

**Figure 3:**
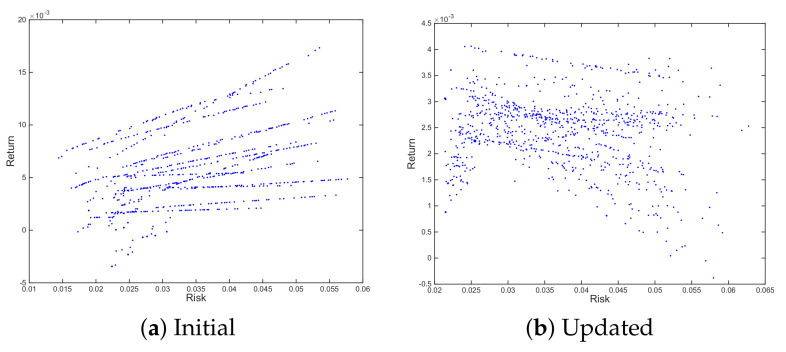
Comparison of initial risk/return profile on 20 resampled frontiers (Initial) vs. average risk/return profile of same portfolios considering 100 scenarios (Updated).

We will overcome the problem of the reliability of the expected risk/return profile of the portfolios obtained in the first stage of the process, exposing them to several scenarios. Once portfolio structures have been set, we will evaluate them for *e* synthetic scenarios and average the resulting risks and returns. This will result in more reliable forecasts for the expected risk and return for the portfolio and, therefore, will be the ones that we will use to fit the polynomial function and to identify the final set. Figure 3 shows the difference between the initial set and the updated one once we consider e=100 scenarios.

**Figure 4:**
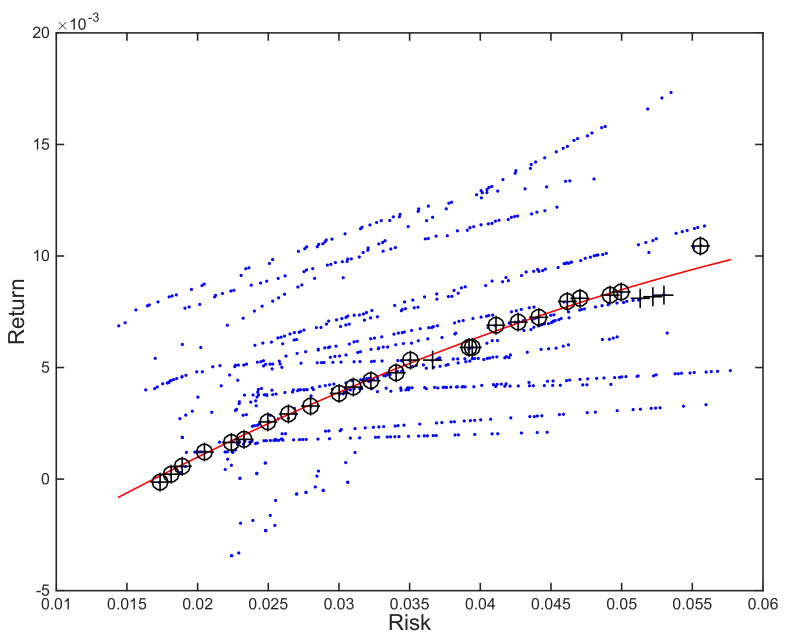
Illustration of approach 2 on 20 resampled frontiers optimized using SPEA2 and an order 2 polynomial trendline. The closest portfolios to the reference points along the polynomial trendline are denoted by +. Final solution includes the non-dominated ones among them, denoted by ⊕.

The process, illustrated in Figure 4, relies on a definition of distance. Given that there are differences in scale, and a relation of proportionality among the two dimensions, we suggest using Mahalanobis distance as the similarity measure.

Mahalanobis distance is widely used to find outliers. Within asset management, Kritzman and Li [25] use it to derive a measure of financial turbulence, which measures the statistical unusualness of a set of returns given their historical pattern of behavior. Boudt et al. [26] rely on it at a more granular level to filter out outliers from the return series that are used to fit prediction models. Stöckl and Hanke [27] also identify a number of related use cases in their study on the financial applications of the Mahalanobis distance. These authors discuss the detection of structural changes over time; the identification of deviations from model prices and checks for arbitrage opportunities, and forecast evaluation.

In this study, our main concern is robustness. We focus our attention on reliability, in the sense of the likelihood of observing a behavior that is similar to the anticipated one. We are interested in the ability to identify sensitive portfolio structures that are subject to extreme deviations caused by estimation errors for the expected asset returns and the variance–covariance matrix.

Risk and return are both correlated and subject to different scales, so we need a multivariate distance metric to characterize deviations from the norm that can cope with these properties. The similarity indicator should also be able to work at the portfolio level. Mahalanobis distance can, therefore, be used to identify sensitive portfolios whose behavior is likely to be extreme, so they can be avoided by the decision maker or, conversely, the most reliable ones, which are the most relevant ones for the suggested approach.

In this case, the distance between risk/reward profile of portfolio *x*, $\left(E\left(Rp_{x}\right),\sigma_{p_{x}}^{2}\right)$ and the reference one along the polynomial trend, *y*, $d_{M}\left(\vec{x},\vec{y}\right)$, is defined as

(6) $$d_{M}\left(\vec{x},\vec{y}\right)=\sqrt{{\left(\vec{x}-\vec{y}\right)}^{T}\Sigma^{-1}\left(\vec{x}-\vec{y}\right)}$$

where $\vec{x}$ and $\vec{y}$ are the patterns to be compared, and Σ is the variance–covariance matrix.

Once again, the upper bound for the number of portfolios that will be included in the final solution will be defined by *v*. As we can see in Figure 4, there might be some portfolios that are dominated and, therefore, excluded from the final solution. In the example, the preselected portfolios are denoted by + sign but, among them, only the ones marked with an additional circle, ⊕, are non-dominated and, therefore, belong to the solution.

As mentioned previously, one of the main advantages of this approach vs. the first one is the fact that all the portfolios included in the final resampled efficient frontier are feasible, regardless of the investment constraints.

### 2.3. Approach 3

**Figure 5:**
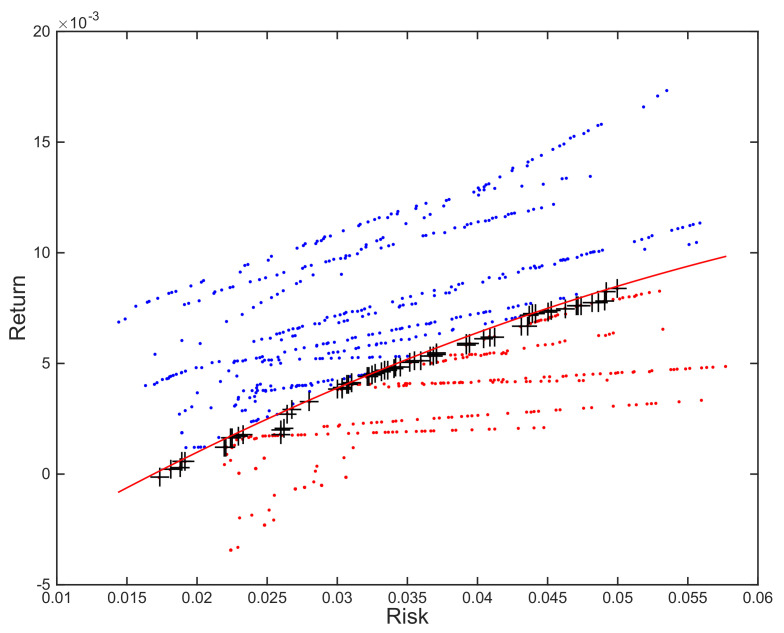
Illustration of approach 3 on 20 resampled frontiers optimized using SPEA2 and an order-2 polynomial trendline. The portfolios included in final solution, denoted by +, include the non-dominated porfolios below the reference trendline (in red).

The third possibility is similar to the previous one. The first steps of the process are the same, but we use a different portofolio selection strategy once we fit the polynomial reference efficient frontier. In this case, we use the frontier to split the portfolio set in two sections: the ones that are over the reference line, and the ones that are below. As we can see in Figure 5, the final estimate for the efficient frontier consists of the non-dominated portfolios, denoted by +, among the second set (in red).

If we compare this alternative with the previous one, it has the advantage of not requiring any similarity measure and the disadvantage of disregarding portfolios that might be right over the reference line to favor others underneath, which might be more distant. However, they both have the advantage of guaranteeing the feasibility of all the portfolios included in the final estimate for the efficient frontier. This property is guaranteed by the fact that both them are based on the selection of portfolios from within a set where all of them are feasible. There is no averaging or transformation of any sort that might be problematic.

Please note that, although we have discussed these approaches in the context of mean–variance portfolio optimization, the core ideas scale with the number objectives. This means that they could be used in more complex settings, like mean–variance–skewness portfolio optimization, provided that we make some adjustments, like replacing polynomial trend lines with trend surfaces.

## 3. Experimental Analysis

In this section, we describe the experimental analysis used to compare the performance of the three alternatives. To that end, we first introduce the sample. This is followed by a description of the process followed to estimate the efficient frontiers for the synthetic scenario. Then, we describe the performance indicators used to evaluate the robustness of the solutions. Finally, we report the experimental design of the results.

### 3.1. Data Set

The dataset that we use in the the experimental analysis consists of eight broad financial indices, sampled monthly over 15 years, from January 2006 to December 2020. The set, reported in Table 1, includes four that cover U.S. equities: large and mid-cap, with either value or growth characteristics, and the equivalent small-cap ones. We also include a broad commodity and and international equity index, and two additional U.S. fixed-income indices: one focused on government bonds and another on corporates. The data were obtained from Refinitiv Eikon.

The dataset used in this work is available for consultation upon request.

### 3.2. Experimental Design and Optimization Process

The evaluation process will start with the definition of a time window of 60 observations that will be used to estimate an efficient frontier for the next one. For instance, we will use the monthly returns for period from January 2006 to December 2010 to forecast an efficient frontier in January 2011. Given that we use a resampling approach, we will optimize it for different scenarios. Each of these will consist of a set expected returns and a variance–covariance matrix obtained from a synthetic return-series-generated sampling vectors of returns from the window with a replacement. In this study, we will consider 20 scenarios.

We will optimize the weights for the eight asset classes with the two constraints mentioned in Section 2: investment limits and cardinality constraints. Regarding the former, the minimum weight for any asset with investment will be 10% and the maximum 80%. As for the latter, out of the eight investment alternatives, a minimum of two and a maximum of six are required in any portfolio.

The optimization will be made using a very popular MOEA, SPEA2. This algorithm, developed by Zitzler et al. [24], is an iteration on SPEA [28] that has been extensively used in finance due to its good properties, such as good convergence to the real Pareto front and spread. SPEA2 is one of the most widely used MOEAs in portfolio optimization according to the references included in the surveys by Metaxiotis and Liagkouras [7] and Ponsich et al. [8], and it is still widely used in this context [29,30].

A comparative study focused on mean–variance portfolio optimization with cardinality constraints by Skolpadungket et al. [31] identified it as the best-performing algorithm among a set that included VEGA [32], a fuzzy version by the authors of the study VEGAFuzl, MOGA [33], NSGA-II [34] and SPEA2, highlighting the quality of the efficient frontiers in terms of generational distance and the distribution along the Pareto front. Anagnostopoulos and Mamanis [35] report similar results. In their article, they evaluate NSGA-II, PESA [36] and SPEA2 in terms of ϵ-indicator and hypervolume on an extended version of the portfolio optimization problem that considered three objectives: risk, return and the number of securities in the portfolio. According to their experimental results, SPEA2 offered the best results for both the constrained and unconstrained multiobjective portfolio optimization problem.

However, the choice serves for illustration purposes. The three approaches presented in this paper are perfectly compatible with the output of any other classic MOEA.

As a very brief introduction, this MOEA uses both a core population and an archive. The fitness valued assigned to the individuals consist of its strength raw fitness, adjusted by a density estimation. The algorithm updates the contents of the archive with the non-dominated individuals of the core population and the existing contents of the archive. Then, it applies the selection, crossover, and mutation evolutionary computation operators to the individuals of the archive to obtain a new set of individuals, which becomes the new population for the next iteration.

The generation of new individuals, which, in this context, consist of vectors of eight reals in the range from zero to one, might result in unfeasible portfolios. This problem also becomes apparent after the use of evolutionary operators. In this situation, we will use two repair algorithms similar to the ones described in [37,38] to transform unfeasible portfolios into feasible ones. The first one, Algorithm 1 was applied after the generation of the initial population.
**Algorithm 1** Repair after initialization.
 Initialize population *P* as a set of vectors with real numbers ${\bar{x}}_{i}=\left(x_{i1},...,x_{in}\right)$; $x_{ij}\in \left[lim_{sup},lim_{inf}\right]$

 **for** each individual ${\bar{x}}_{i}$ of *P*
**do**
  A random number ∈$\left[C_{min}-1,n-C_{max}\right]$ of values 0 are assigned to coordinates of vector ${\bar{x}}_{i}$
  **while**
$\Sigma_{j=1}^{n}x_{ij}\neq 1$
**do**
   **if** sum of the $lim_{inf}$ of $x_{ij}\neq 0$ is >1
**then**
    select random coordinate *j* of vector ${\bar{x}}_{i}$ such that $x_{ij}\neq 0$ and assign $x_{ij}=lim_{inf}$
   **end if**

   **if** sum of the $lim_{sup}$ of $x_{ij}\neq 0$ is <1
**then**
    select random coordinate *j* of vector ${\bar{x}}_{i}$ such that $x_{ij}=0$ and assign $x_{ij}=lim_{sup}$
   **end if**
   **if**
$\Sigma_{j=1}^{n}x_{ij}\neq 1$
**then**

    select random coordinate *j* of vector ${\bar{x}}_{i}$ such that $x_{ij}\neq 0$
    add/subtract the quantity left to make $\Sigma_{j=1}^{n}x_{ij}=1$ meeting $\left[lim_{inf},lim_{sup}\right]$
   **end if**

  **end while**

**end for**

Return(*P*)


The generation of the initial population starts by assigning random weights to all the assets. At that point, the first step is making sure that the cardinality constraints are met. To that end, we drop a random number of assets that ensure that the amount of non-zero weights is in the range between the allowed minimum and the maximum. Then, the non-zero weights are forced to comply with the investment limits [liminf,limsup] and, finally, the algorithm finetunes the holdings, either increasing or decreasing the weights of random assets to adjust for either excess or insufficient investment.

The second, Algorithm 2, was used on the output of genetic operators. As we can see, it is based on the same principles and operates in a similar fashion.
**Algorithm 2** Repair after genetic operators.
 **for** each individual ${\bar{x}}_{i}$ of *P*
**do**
  **if** there are fewer $x_{ij}=0$ than $C_{min}$
**then**
   set one random $x_{ij}=0$ to $x_{ij}=lim_{inf}$
   **end if**
   **if** there are more $x_{ij}=0$ than $C_{max}$
**then**
    set the $x_{ij}\neq 0$ with lowest value to $x_{ij}=0$
  **end if**

  **while**
$\Sigma_{j=1}^{n}x_{ij}\neq 1$
**do**

   **if** sum of the $lim_{inf}$ of $x_{ij}\neq 0$ is >1
**then**
    select random coordinate *j* of vector ${\bar{x}}_{i}$ such that $x_{ij}\neq 0$ and assign $x_{ij}=lim_{inf}$
   **end if**

   **if** sum of the $lim_{sup}$ of $x_{ij}\neq 0$ is <1
**then**
    select random coordinate *j* of vector ${\bar{x}}_{i}$ such that $x_{ij}=0$ and assign $x_{ij}=lim_{sup}$
   **end if**

   **if**
$\Sigma_{j=1}^{n}x_{ij}\neq 1$
**then**

    select one random $x_{ij}\neq 0\in \left[lim_{inf},lim_{sup}\right]$
    add/subtract one random quantity left to make $\Sigma_{j=1}^{n}x_{ij}=1$ meeting $\left[lim_{inf},lim_{sup}\right]$
   **end if**

  **end while**

 **end for**

Return(*P*)


The main parameters used are reported in Table 2.

The output of this process is 20 estimates for the efficient frontier on January 2011. Then, we use the three approaches to combine these into three different resampled efficient frontiers and evaluate their robustness using the metrics described in the next subsection. The number of scenarios used to update the expected risk and return for the set of portfolios obtained by optimizing for the synthetic scenarios is e=100 in both approaches 2 and 3.

At this point, we shift the time window one month, and obtain the resampled efficient frontiers and the metrics for February 2011. The process is repeated until we obtain the results for December 2020 and, finally, we will compare the performance of the three strategies over the 120 periods.

### 3.3. Stability Measures

Given that the aim of the process is increasing the robustness of the estimates, we will evaluate the relative performance of the three approaches on the grounds of general stability and sensitivity to extreme scenarios. These indicators, already used in studies like [20], are described below.

- **Estimation Error**: this indicator measures the aggregate sensitivity of the forecast for efficient frontier to a large number of probable scenarios, regardless of the one which happened to materialize. It is computed by averaging the mean average deviation in the portfolio’s expected risk/return profiles vs. the expected ones over *S* different synthetic scenarios generated using non-parametric bootstrap. Once again, given the scale differences and the connection between the two objectives, we rely on Mahalanobis distance. The metric is formally defined as
  (7) $$ST=\frac{1}{S}\sum_{i=1}^{S}\frac{1}{N}\sum_{p=1}^{N}d_{M}{\left(\vec{x_{p}},\vec{x_{pi}}\right)}^{2},$$
  where $d_{M}\left(\vec{x_{p}},\vec{x_{pi}}\right)$ is the Mahalanobis distance between $\left(E\left(R_{np}\right),\sigma_{np}^{2}\right)$, the expected return and risk of portfolio *p* in period $t_{n}$ and $\left(E\left(R_{npi}\right),\sigma_{npi}^{2}\right)$, the risk and return of the same portfolio and period, for the synthetic scenario *i*.

Higher values of this metric represent lower robustness, as they indicate higher average dispersion and, therefore, lower reliability of the estimates for the expected behavior. In this study, we will use S=500 different scenarios generated using the sampling process already described in Section 2.

- **Sensitivity to Extreme Risk**: This metric evaluates the impact of worst-case scenarios on the reliability of the forecasted efficient frontier. This is a constrained version of the previous one, focused on the *w* worst scenarios. In this case, the same *S* scenarios are generated and evaluated, but the computation of the mean average Mahalanobis distance is limited to the scenarios for which this deviation is the largest. This serves as a tail risk indicator that measures the expected average negative outcome of the realization of the worst scenarios with probability w/S. Once again, the higher the metric, the less robust the solution. In the experimental analysis, we will consider two thresholds: the 5% worst scenarios and the 1% ones.

### 3.4. Experimental Results

The main experimental results are reported in Table 3. There, we provide the main descriptive statistics for the robustness indicators by approach. For each of the latter, we report the mean, median, standardad deviation, minimum and maximum value for the three performance indicators (stability and sensitivity to extreme risks at 5% and 1%). These indicators were computed for the 120 consecutive months between January 2011 and December 2020 based on the immediate previous 60.

Regarding these approaches, we include the main three introduced in Section 2, plus a variation on the first one that uses the reconstruction operator described in Algorithm 2, which we designate *Approach 1 (R)*. The operator was applied to all the unfeasible portfolios included in the resampled efficient frontier obtained from Approach 1. Given that this reconstruction process sometimes produces dominated portfolios, we added a filtering step to make sure that none of them were included in the final solution.

If we consider stability, approaches 2 and 3 consistently beat the first one in terms of both mean and median results. Approach 2 seems to be marginally better, but the difference compared to the third one is very small. It is worth noting that both approaches 2 and 3 present lower maximum and minimum values.

The two procedures that select portfolios from the set also seem to be superior to the averaging strategy when it comes to limiting the impact of harmful scenarios. Regarding the reliability of the estimate on the *w* worst-case scenarios, we find the same pattern that we identified for stability. However, this time the third approach beats the second one by a very small margin. The first one is the worst in mean and median terms, but it provides the best best-case scenario, with the lowest average minimum deviation. We observe this regardless of whether we consider the worst 5% out of the 500, or we focus on the most damaging 1%.

The impact of the reconstruction operator on the first approach is very limited. According to the experimental results summarized in Table 3, depending on the metric and whether we use the average or the median, one outperforms the other or vice versa. There seem, however, to be relevant disparities in the reliability or the two. The reconstruction operator guarantees the feasibility of all portfolios included in the resampled efficient frontier, which is a major advantage, but it comes at the price of a higher variance. Still, these differences do not affect the dominance of approaches 2 and 3.

The statistical significance of the differences reported in Table 3 is also formally tested. Given the lack of normality, we follow the protocol suggested in [39] and we start considering the global set approaches with Friedman’s test [40]. Once we rejected the null hypothesis, we proceeded with Shaffer’s static procedure [41] for the post-hoc test of the pairwise differences using the implementation in KEEL 3.0 [42].

As we can see in Table 4, the null hypothesis of no difference among the four approaches is rejected at the 1% confidence level for the three robustness indicators. Hence, there is a need to study the pairwise differences. The results of this analysis are summarized in Table 5.

As we can see, the outcome of the post-hoc comparisons using Shaffer’s static procedure shows the same pattern across the three robustness metrics. The differences among the first approach and the extended version with the reconstruction operator are not significant enough to reject the null hypothesis of equality at 5%. We see the same when we compare the performance of the second and the third approaches. However, the tests confirm the dominance of the last two at 1%.

In order to assess the relative computational cost of the three alternatives, we timed them using a Matlab implementation on a 10th Gen 2GHz Intel Core i5 CPU and 16 GB of 3733 MHz LPDDR4X RAM. The average times over the five experiments required by the first, second and third approaches were 3.55 s, 197.03 s and 204.21 s, respectively. Out of the last two, 98% of the time for the second and 95% for the third one was spent updating the expected risk and return over the e=100 synthetic scenarios, as described in Section 2.2. Regarding the version that uses the reconstruction operator and filters out dominated portfolios, the average increased to 4.78 s due to the additional overhead.

For comparison purposes, we evaluated the original MM resampled efficient frontier. This required a combination quadratic programming and brute-force on the combinatorial side of the problem to guarantee the mentioned properties required by the averaging process.

This was only possible because the problem used to illustrate the approaches considers a very limited number of assets. Once we increase this, the exhaustive search of the combination of assets in the range required by the cardinality constraint and the weight optimization becomes so costly that we are forced to rely on heuristic alternatives, like MOEAs. Hence, the solution strategies introduced in this work are important. As we discussed in the introduction, if we consider a large investment universe, the process described by MM becomes unfeasible in practice and comparisons like this one are not an option.

It is also worth emphasizing, once again, the fact that the comparison is not completely fair, as the averaging process of the standard MM approach results in unfeasible portfolios. Even though the full investment constraint is guaranteed to be met, violations of the boundaries on the minimum and maximum weights and the cardinality limits are not controlled at any time during the process.

In order to gain more insight into the connection between the structure of the efficient frontiers for the synthetic scenarios and the final results, we also analyzed the performance that we obtained by testing the three approaches, and the variation on the first one, on the efficient frontiers for the synthetic scenarios obtained by brute-force and quadratic programming.

The results, summarized in Table 6, show that the standard MM approach offers competitive robustness indicators among the best of the five alternatives. If we consider median robustness, it is always one of the best two, and it also provides the lowest maximum values across the board.

Regarding the three strategies introduced in this work, we observe the superiority of the second and the third over the first one. One might consider stability to be an exception, but the advantage of the first approach in the mean results disappears once we focus on the medians. The reconstruction operator turned out to be very destructive, and using it with these inputs degrades the performance of the first approach very significantly in terms of both mean and median.

Interestingly, the differences between the performance of the canonical approach and the second and third approach, formally tested in Table 7 and Table 8, are small. This suggests that the dominance of the MM approach over the ones based on SPEA2 is likely to mostly be explained by the advantage of having perfectly regular efficient frontiers as the input for the process.

Once again, the Friedman test rejects the null hypothesis of the non-existence of differences among the five alternatives at the 1% confidence level for the three robustness indicators.

The post-hoc test of the pairwise differences offers interesting conclusions. While the vast majority of them are significant at 1%, we find two exceptions: the performance of the first and second approaches and, more interestingly, the solution based on the canonical MM approach vs. the third one.

These experimental results support the idea that the robustness of the resampled efficient frontier provided by the all integration strategies is directly related to the quality of the efficient frontiers for the synthetic scenarios used as inputs. However, the third approach has the advantage of offering good performance vs. the alternatives with standard quality inputs, and benefits a great deal from the high-quality ones. According to these results, this happens to the point of offering a performance that is comparable to the one resulting from the MM process in two out of the three robustness indicators, and is better in the third one. This suggests future advances in the field of MOEAs that result in regular Pareto fronts will directly translate into more reliable estimates for the efficient frontiers through the use of the approaches discussed in this study.

To summarize, approaches 2 and 3 are competitive in terms of robustness with the averaging alternative put forward by MM, while, at the same time, offering two major advantages: guaranteeing compliance with portfolio constraints, and offering an alternative that is agnostic to the algorithm used to optimize the synthetic scenarios. Among these two, the third one is especially promising, as the experimental results support the idea that it benefits from better quality inputs to a greater extent.

## 4. Summary and Conclusions

Mean–variance portfolio optimization is subject to uncertainty issues related to the reliability of the estimates of input parameters for the optimization problem. The estimation process of the efficient frontier requires accurate forecasts for the future returns and the variance–covariance matrix. This means that, in practice, the real risk/return profile of the portfolios identified in the process might be very different from the expected one.

A popular way of tackling this problem is using resampling strategies where one defines a number of synthetic scenarios based on real data, computes the efficient frontiers for all of them, and combines the resulting set of solutions into a single one. This results in a more robust estimate, as it is not optimized for a single expected scenario, but it also includes information about many other likely ones.

This combination problem, averaging the structure of those portfolios that either minimize risk for the same rank within sets of equispaced returns, or maximise utility for the same risk aversion, is not an option if the optimization problem is solved using many standard mutiobjective evolutionary algorithms. The output of these is likely to consist of a variable number of portfolios, which are often unevenly spread in terms of both return and risk aversion, hence the need to adapt the process to make it compatible.

In this work, we introduced three possibilities to adapt the MM approach, which use a polynomial function as an initial reference for a potential estimate for the efficient frontier. This function is fitted on a set of efficient frontiers optimized for synthetic scenarios and, depending on the approach, will either be used to select subsets of portfolios to be combined, or portfolios to be directly included in the final solution. The output of the former suffers from the limitation that it might include unfeasible portfolios in the constrained versions of the problem. This is also the case in the standard approach proposed by MM. The other two, however, are free from this limitation.

The experimental results for the output of a popular MOEA, SPEA2, support the superiority of the approaches based on portfolio selection over the one which relies on portfolio averaging. The former outperformed the latter on the three considered robustness metrics, and the use of a reconstruction operation in the output of the averaging process did not have a significant impact on the quality of the solutions. Regarding the other two, favoring portfolios that were close to the reference efficient frontier resulted in slightly higher stability than choosing the non-dominated portfolios below it. Conversely, the results were the opposite for sensitivity to extreme risks.

In order to obtain a better understanding of the abilities of these approaches, the problem was also tackled using an exhaustive exploration of the potential asset combinations and quadratic programming to identify the optimal portfolio weights. Based on the efficient frontiers for the synthetic scenarios, we estimated the resampled efficient frontier as per MM, the core three approaches presented in this study, and the variation of the first one, which uses reconstruction.

The evaluation of these results suggests that the standard averaging approach outperforms the alternatives based on MOEAs due to the balanced structure of the efficient frontiers required as inputs. Once we use these high-quality inputs with the new integration approaches, we obtain a similar degree of robustness. In fact, when we compared the third one with MM, we could not reject the null hypothesis of equality at 1% for two out of the three robustness indicators. In the case of third one, the solution introduced in this study outperformed Michaud and Michaud.

The three approaches introduced in this work pose a significant contribution, as they introduce the possibility of more robust estimates for efficient frontiers using standard MOEAs. Unlike previous alternatives found in the literature, these are based on the canonical process by MM and do not require any adaptation to either the fitness function or the core algorithm. This ease of implementation makes the solutions accessible to a wider audience.

The two that are based on portfolio selection are competitive when compared to MM, while, at the same time, they guarantee compliance with portfolio constraints. They are also flexible in terms of the structure of the efficient frontiers for the synthetic scenarios used as inputs. In this regard, the experimental results also suggest that they will benefit from any progress in the field of MOEAs, which leads to more regular solutions without further adaptation.

Future extensions of this work include adjusting the approaches and evaluating their performance in settings with more than two objectives, exploring new alternatives based on clustering, or the analysis of the risk aversion of the set of portfolios derived from the synthetic scenarios, and replicating the study on new datasets. There also seem to be promising lines of work identifying trading strategies based on estimates for the efficient frontier and testing whether this additional robustness effectivley contribute to improving financial performance.

## Figures and Tables

**Table 1:** Data set.

Name	Code
Frank Russell 2000 Growth	FRUS2GR
Frank Russell 1000 Value	FRUS1VA
Frank Russell 1000 Growth	FRUS1GR
S&P GSCI Commodity Total Return	GSCITOT
MSCI EAFE	MSEAFEL
S&P U.S. Treasury Bond Index	SPBDUSB
IBOXX $ Corporates	IBUCPAL

**Table 2:** SPEA2 optimization parameters.

Parameter	Value
*Generations*	1000
*Population size*	50 individuals
*Archive size*	50 individuals
*Crossover*	SBX, $p_{c}$ = 0.7
*Mutation*	Polynomial, $p_{m}$ = 1/L
*Selection of Parents*	Binary tournament

**Table 3:** Descriptive statistics of robustness indicators by approach based on SPEA2. Indicators include stability and sensitivity to extreme risks at 5% and 1%. Results for 20 scenarios and monthly returns over the period from January 2011 to December 2020.

		Mean	Median	St. Dev.	Min	Max
Stability	Approach 1	0.9502	0.9451	0.0809	0.8241	1.2849
	Approach 1 (R)	0.9515	0.9268	0.0978	0.8243	1.2849
	Approach 2	0.8817	0.8611	0.0582	0.8035	1.0957
	Approach 3	0.8890	0.8642	0.0803	0.7845	1.1782
ER 5%	Approach 1	2.3048	2.3126	0.1645	1.2776	2.6757
	Approach 1 (R)	2.2911	2.3048	0.2159	1.2564	2.6378
	Approach 2	2.2474	2.2447	0.0866	2.0589	2.5179
	Approach 3	2.2432	2.2390	0.1063	2.0362	2.5562
ER 1%	Approach 1	2.8026	2.8091	0.2553	1.2776	3.2984
	Approach 1 (R)	2.7820	2.8116	0.3264	1.2564	3.2297
	Approach 2	2.7506	2.7312	0.1680	2.4015	3.1824
	Approach 3	2.7418	2.7189	0.1810	2.3960	3.1863

**Table 4:** Average ranks by approach, obtained by applying the Friedman procedure on the results reported in Table 3.

	Stability	ER 5%	ER 1%
Approach 1	3.3083	3.2160	3.1750
Approach 1 (R)	3.1333	3.0542	3.0833
Approach 2	1.6917	1.9667	1.9667
Approach 3	1.8667	1.7625	1.7750
Friedman Statistic	151.8500 ^++^	118.7325 ^++^	115.6300 ^++^

++ Difference of medians significant at 1%.

**Table 5:** Statistical significance of differences in medians reported in Table 3. Results on post-hoc comparisons using Shaffer’s static procedure.

	Stability				ER 5%		
	Ap. 1 (R)	Ap. 2	Ap. 3		Ap. 1 (R)	Ap. 2	Ap. 3
Ap. 1	=	++	++		=	++	++
Ap. 1 (R)		++	++			++	++
Ap. 2			=				=
	ER 1%		
	Ap. 1 (R)	Ap. 2	Ap. 3
Ap. 1	=	++	++
Ap. 1 (R)		++	++
Ap. 2			=				

++ Difference significant at 1%; = Equality cannot be rejected at 5%.

**Table 6:** Descriptive statistics of robustness indicators for solutions based on brute-force combinatorial optimization using quadratic programming. Results for the standard Michaud and Michaud resampling process and the three novel approaches. Indicators include stability and sensitivity to extreme risks at 5% and 1%. Results for 20 scenarios and monthly returns over the period from January 2011 to December 2020.

		Mean	Median	St. Dev.	Min	Max
Stability	Standard	0.7546	0.7475	0.0267	0.7182	0.8486
	Approach 1	0.7670	0.7713	0.0379	0.6184	0.8705
	Approach 1 (R)	0.8367	0.8194	0.0992	0.6828	1.2773
	Approach 2	0.8148	0.7660	0.1389	0.6892	1.3001
	Approach 3	0.7949	0.7395	0.1619	0.5583	1.2573
ER 5%	Standard	1.9933	1.9915	0.0728	1.8294	2.1928
	Approach 1	2.0154	2.0362	0.1267	1.6319	2.2894
	Approach 1 (R)	2.1359	2.1557	0.1847	1.2636	2.6178
	Approach 2	1.9858	2.0306	0.2172	1.2387	2.5159
	Approach 3	1.9936	1.9468	0.2662	1.2681	2.8748
ER 1%	Standard	2.4472	2.4462	0.1447	2.1412	2.8010
	Approach 1	2.4725	2.4789	0.1785	2.0538	2.8655
	Approach 1 (R)	2.6046	2.6124	0.2698	1.2636	3.2084
	Approach 2	2.4109	2.4645	0.3488	1.2387	3.0522
	Approach 3	2.4240	2.3671	0.3116	1.6379	3.6034

**Table 7:** Average ranks by approach obtained applying the Friedman procedure on the results reported in Table 3.

	Stability	ER 5%	ER 1%
Standard	2.0750	2.3500	2.4080
Approach 1	3.0417	3.0500	3.0580
Approach 1 (R)	4.5583	4.5500	4.5500
Approach 2	3.3167	3.0920	3.1000
Approach 3	2.0083	1.9580	1.8830
Friedman Statistic	209.7333 ^++^	118.2067 ^++^	192.6200 ^++^

++ Difference of medians significant at 1%.

**Table 8:** Statistical significance of the differences in medians reported in Table 6. Results of post-hoc comparisons using Shaffer’s static procedure.

	Stability					ER 5%			
	Ap. 1	Ap. 1 (R)	Ap. 2	Ap. 3		Ap. 1	Ap. 1 (R)	Ap. 2	Ap. 3
Standard	++	++	++	=		++	++	++	=
Ap. 1		++	=	++			++	=	++
Ap. 1 (R)			++	++				++	++
Ap. 2				++					++
	ER 1%			
	Ap. 1	Ap. 1 (R)	Ap. 2	Ap. 3
Standard	++	++	++	+
Ap. 1		++	=	++
Ap. 1 (R)			++	++
Ap. 2				++					

++ Difference significant at 1%; + Difference significant at 5%; = Equality cannot be rejected at 5%.

## Data Availability

Not accplicable.
